# Efficacy of an Individualized Computer-Assisted Social Competence Training Program for Children With Oppositional Defiant Disorders/Conduct Disorders

**DOI:** 10.3389/fpsyt.2019.00682

**Published:** 2019-09-18

**Authors:** Anja Goertz-Dorten, Manuela Groth, Kerstin Detering, Anne Hellmann, Laura Stadler, Barbara Petri, Manfred Doepfner

**Affiliations:** ^1^Department of Child and Adolescent Psychiatry, Psychosomatics and Psychotherapy, Medical Faculty at the University of Cologne, Cologne, Germany; ^2^School of Child and Adolescent Behavior Therapy at the University Hospital Cologne, Cologne, Germany; ^3^Institute of Child and Adolescent Psychotherapy of the Christoph-Dornier-Foundation for Clinical Psychology at the University of Cologne, Cologne, Germany

**Keywords:** disruptive, impulse control, and conduct disorders, cognitive behavioral therapy, social skills, therapy, computer-assisted, treatment outcome

## Abstract

Group-based child-centered cognitive behavioral therapy (CBT) for children with aggressive behavior has been found to significantly reduce child behavior problems. Nevertheless, most children suffer from residual symptoms at the end of treatment. Therefore, individualized interventions that treat the specific problem-maintaining factors and that use digital support may enhance treatment effects. However, enhanced computer-facilitated interventions have not been examined in clinical samples. Therefore, we tested the efficacy of an individualized computer-facilitated social skills training for children with clinically referred aggressive behavior problems. Fifty children aged 6–12 years with peer-related aggressive behavior problems were included in a within-subject design with two phases (waiting, treatment). The course of the outcome measures during an 8-week waiting phase was compared with that in the subsequent treatment phase (16 weekly child sessions and 2 parent psychoeducation contacts at the beginning of the treatment) using multilevel modeling. The primary outcome was peer-related aggressive behavior rated by parents. Further outcome measures included parent ratings and patient self-reports of aggressive and prosocial behavior. No significant changes occurred for any of the outcome variables during the waiting phase. During treatment, most parent-rated outcome measures (including the primary outcome measure) showed a significant decrease, which was stronger than changes in the waiting phase. Most self-rated outcome measures also showed significant decreases during treatment, but a stronger decrease than in the waiting phase was only found for peer-related aggressive behavior. The computer-facilitated social skills training appears to be an effective CBT intervention for children with peer-related aggressive behavior.

## Introduction

Aggressive and oppositional behavior problems in children are widespread, with prevalence rates ranging from 1% to 11% for oppositional defiant disorders (ODDs) and from 2% to 10% for conduct disorders (CDs). Moreover, aggressive behavior problems often persist from childhood to adolescence (1), and children with early aggressive problem behavior have a higher risk of adverse developmental outcomes in adolescence and adulthood, such as ongoing mental health problems, academic underachievement, and substance use [e.g., Refs. (2, 3)]. Aggressive and oppositional problem behavior in children can be directed toward adults (e.g., parents, teachers) or peers. Peer-related and peer-reported aggression is important because it is a better predictor of maladaptive outcomes in late adolescence and early adulthood than parent and teacher ratings of oppositional–aggressive behavior (4).

Parent training has been shown to be effective in the treatment of children with ODD/CD (5). Moreover, positive effects of child-based interventions on children’s social skills in their interactions with peers have been demonstrated [e.g., Ref. (6)]. Child-based social skills training may be the treatment of choice for modifying peer-related aggressive behavior because the child can be trained directly, and parents or other adults are not necessarily present when conflicts with peers occur. However, research over the past three decades suggests that child-based treatment has only modest effects on aggressive behavior (5, 7).

One of the main drawbacks of such group-based interventions may be that they are not individually tailored to address the specific problem-maintaining factors for each child (8). Moreover, evidence suggests that the practice of grouping children for the purpose of teaching and practicing social skills may lead to unanticipated outcomes such as unintended changes in attitudes to antisocial behavior, identification with deviant peers, and assimilation of deviant values (9–11). Recently, studies have demonstrated the efficacy of individually tailored interventions on peer-related aggressive behavior (12, 13), supporting the hypothesis that social skills training in an individual setting can be more effective in reducing child aggressive behavior problems than group-based interventions. In these analyses, we found moderate to strong effects of an individualized treatment that focuses on the reduction of the individual problem-maintaining factors (i.e., social cognitive information processing, impulse control, social problem solving, and/or social skills) in specific conflict situations of the individual child.

Moreover, computerized cognitive behavioral therapy (cCBT), may enhance treatment effects through the integration of technological support into face-to-face treatment.

Although some of the classical social skills training interventions incorporate video vignettes [e.g., Refs. (14, 15)], more refined computer-facilitated interventions have not yet been examined in clinical samples (16). A small number of preventive approaches employ cCBT (e.g., Zoo U online game) (17) to strengthen social and emotional skills for success in the classroom and in everyday life, or incorporate video modeling (e.g., TD Social Skills—video modeling DVD series; http://www.tdsocialskills.com) to increase and generalize social skills. A study by Carrol et al. (18) investigated the effects of video-based vignettes related to classroom social behavior on attention and comprehension of social behaviors in children with attention deficit/hyperactivity disorder (ADHD) and found empirical support for the use of technology-supported social skills interventions. Fenstermacher et al. (8) assessed the effectiveness of a computer-facilitated, interactive social skills training program for boys with ADHD in a multiple baseline design with a small sample of four children. All participants showed improvements in social problem-solving skills during analogue role-play assessments with peers.

Thus, while computer-facilitated social skills training for children with aggressive behavior problems is promising, it has not yet been evaluated in larger clinical samples. Therefore, we investigated the efficacy of a computer-assisted social competence training program for children with aggressive behavior (ScouT) (19) in a clinical sample of children with ODD/CD and peer-related aggression. ScouT is an individualized, rather than group-based, social competence training program for children aged 6–12 years. It was specifically developed to change peer-related aggressive behavior that causes persistent impairment of relationships with other children. Moreover, the intervention aims to treat individual problem-maintaining and moderating factors of aggressive behavior in specific daily life situations that each child has experienced in previous weeks. Depending on the individual problem-maintaining factors, ScouT aims to modify social cognitive information processing, impulse control, social problem solving, and/or social skills in these specific situations. ScouT presents short video vignettes of typical peer-related conflict situations, with different reactions on a cognitive, emotional, and behavioral level, and with different social consequences. These video vignettes are combined with animated cartoons and specific interactive questions and reinforcement. We expect that this technological support will help the patient to detect his/her own deficits in social skills and to train a socially competent mastery of such conflicts. Moreover, we expect this form of presentation to be more stimulating and motivating for the child, thus possibly resulting in an enhanced outcome.

The present study analyzes the efficacy of the treatment on various outcomes including ODD symptoms, CD symptoms, and prosocial behavior, as well as problem-maintaining and moderating factors of aggressive behavior toward peers and adults rated by parents and patients. We compared the course of these outcome measures during a waiting phase with the course during treatment in a within-subject controlled design. Specifically, we expected to find a stronger reduction in symptoms and problem-maintaining and moderating factors, as well as a stronger improvement in prosocial behavior during the treatment phase compared to the preceding waiting phase.

## Methods

### Study Design

This analysis, which assesses the effects of the treatment with ScouT in comparison to a preceding waiting phase, is part of a larger clinical trial. The study protocol (ClinicalTrials.gov identifier: NCT02143427) was approved by the ethics committee of the University Hospital Cologne. The process of checking for eligibility included an 8-week waiting phase, following which eligible children received treatment with ScouT. Data were collected at three assessment points: (1) pre1 (at the beginning of the 8-week waiting phase), (2) pre2 (at the end of the 8-week waiting phase and immediately before the start of the 16-week treatment phase), and (3) post (at the end of the intervention).

### Study Recruitment and Inclusion Criteria

Families were recruited in an urban area in Germany (Cologne) *via* cooperation with outpatient units and private practices for child and adolescent psychiatry or child and adolescent psychotherapy, youth welfare offices, schools, and the media. Most of the patients were treatment-naïve, and parents or teachers were seeking the treatment. Children were included if they were aged 6–12 years with an IQ ≥ 80 according to the Culture Fair Intelligence Test (20, 21) and if they fulfilled criteria for an International Classification of Diseases, 10th Revision (ICD-10) diagnosis associated with aggressive behavior problems (F91: CD including ODD; F92: mixed disorder of conduct and emotions; or F90.1: hyperkinetic conduct disorder), assessed *via* a semi-structured interview for ODD and CD with the Diagnostic Checklist for Disruptive Behavior Disorders (DCL-DBD) of the German Diagnostic System for Children and Adolescents (DISYPS-II) (22). The ICD-10 diagnoses correspond to *Diagnostic and Statistical Manual of Mental Disorders, Fifth Revision* (*DSM-5*), diagnoses of CD, ODD, and CD/ODD plus ADHD. Furthermore, children had to show peer-related aggressive behavior causing persistent impairment of relationships with other children (clinical rating on the basis of a semi-structured interview) and a high total score (Stanine ≥ 7) in parent rating on the Symptom Checklist for Disruptive Behavior Disorder (SCL-DBD) of the DISYPS-II (22) before and after the waiting phase. Exclusion criteria were a primary comorbid disorder according to clinical judgment (e.g., autism), a planned change of medication in children receiving psychotropic medication, current psychotherapy of the child, and severe mental disorder of the participating parent. Parents and children gave their informed consent for inclusion in the study after the procedure had been fully explained. No incentives were given for taking part in the trial.

### ScouT Treatment

The computer-assisted social competence training for children with aggressive behavior (ScouT) (19) is a computer-assisted program to train social problem-solving skills for children with aggressive behavior problems aged 6–12 years. It comprises a therapist manual and an interactive DVD. ScouT was developed for children who show aggressive behavior especially toward peers. In a stepwise approach, children learn cognitive, emotional, and behavioral skills to adequately solve peer conflicts without the use of aggressive behavior. The training is theoretically based on the model of social information processing (23, 24), according to which aggressive behavior is influenced by one or multiple deficits in social information processing, impulse control, social problem solving, or social skills.

ScouT integrates elements of traditional social skills training and applies various cognitive behavioral methods (e.g., overt/covert modeling, coping modeling, mastering modeling, vicarious reinforcement) *via* video films and animated cartoon characters. Additional individual role-plays between the therapist and patient using puppets, including feedback from the therapist, help the child to adapt socially competent behavior to conflict situations he/she has experienced in his/her real life. Therapeutic homework assignments support the transfer of socially competent conflict solutions to the real-life setting.

ScouT includes video vignettes of five peer-related conflict situations in which the protagonist is confronted with (1) disappointment, (2) verbal aggression, (3) physical aggression, (4) non-acceptance of responsibility, and (5) depreciation. ScouT assists the therapist and the child by asking specific questions that help to explore the patient’s social problem-solving skills and deficits and to modify them in a second step.

The short stories are told from the perspective of the main character and the interaction partner, respectively. The video sequences start by demonstrating the conflict situation, which helps the child to identify similar experiences of his/her own with peers in the past. The child first has the task of describing what happened in the video sequence in such a situation.

Following each film, four alternative solutions for the conflict situations are presented (socially competent, socially unassertive, verbally aggressive, physically aggressive). Internal dialogues of the characters are added in order to provide insight into the characters’ appraisal of the situation or to illustrate feelings of the main character and the interaction partner. The patient is asked to choose the solution that describes best how he/she would think, feel, or act as the protagonist.

Afterward, the child and the therapist watch the video with the alternative chosen by the child. The child is then asked to identify the thoughts and feelings of both involved characters and to describe the possible consequences of the behavior. Finally, the child is asked to identify similar interactions in his/her real life.

The child can then watch further sequences that show how the situation evolves and which consequences follow the behavior of the protagonist. The child identifies the best solution in the specific conflict situation (*What is the best solution?*) and is asked to transfer it to a real problem situation that the child has experienced in the past.

In the current study, ScouT included 16 weekly child sessions (lasting 50 min each) and 2 psychoeducation sessions with parents. The training was conducted by 11 experienced child therapists, who received weekly group supervision from a senior child therapist (AG-D, first author of the study).

### Participants and Treatment Assignment

**Figure 1:**
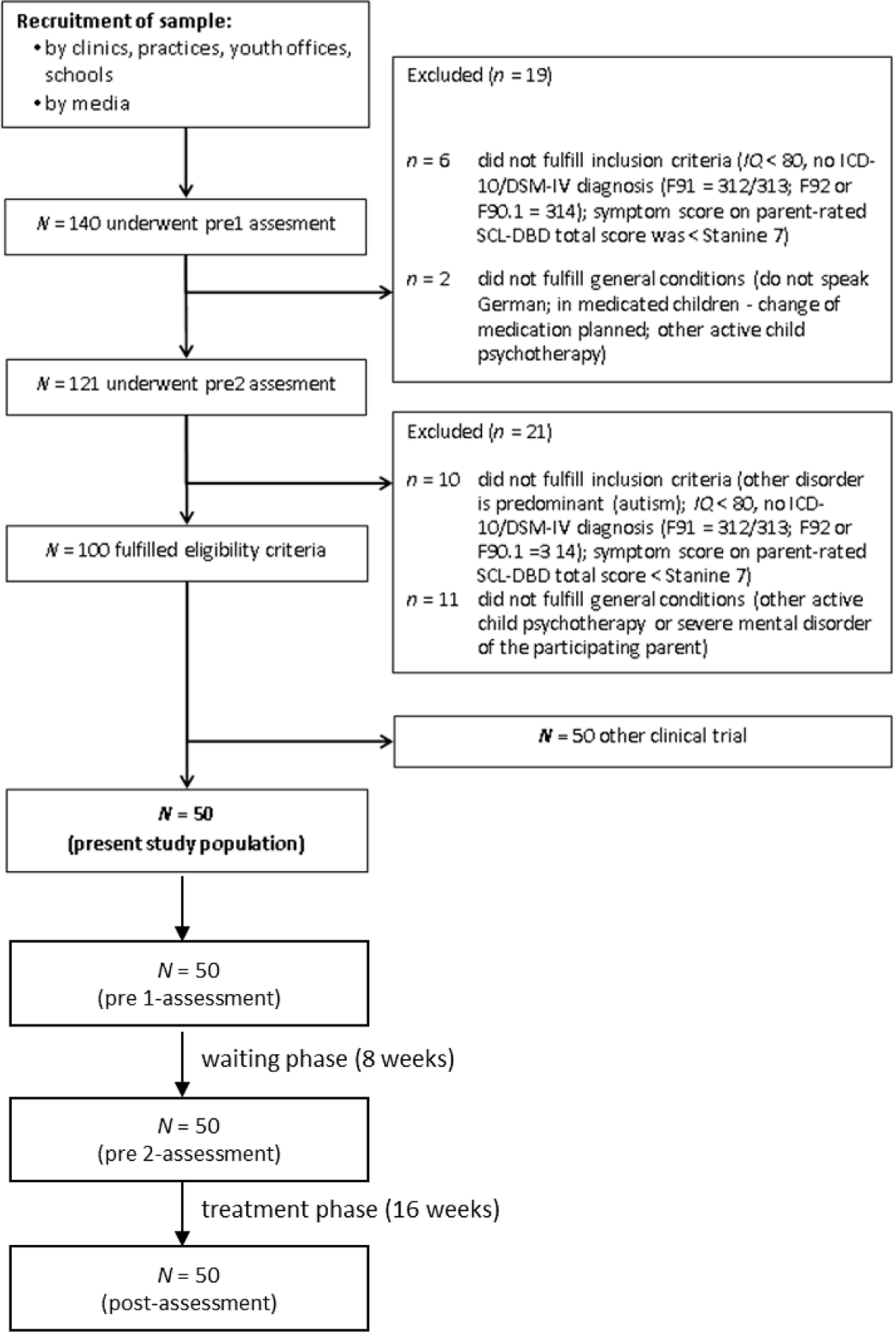
Flow of participants through the study.

**Figure 1** shows the flow of the participants through the study. A total of 140 patients and their parents were assessed for eligibility at the pre1 assessment. Of these, 19 patients were excluded at the pre1 assessment. Of the 121 patients and their parents who supplied questionnaire data at the pre2 assessment, a further 21 patients did not fulfill the inclusion criteria. From the remaining 100 patients, 50 patients were entered into the ScouT group, while the other 50 patients were randomly selected for another clinical trial. The present analysis reports on the 50 patients in the ScouT group.

The patients (92% male) had a mean age of 9.12 years (*SD* = 1.79) and were 98% Caucasian and 2% black. Thirty-two percent of the patients had an immigration background, defined by at least one parent/grandparent being born outside of Germany. The distribution of ICD-10 diagnoses was as follows: 12% conduct disorder (F91.1), 6% F91.2, 66% oppositional defiant disorder (F91.3), and 16% hyperkinetic conduct disorder (F90.1). Diagnoses according to *DSM-IV* were: 20% CD (312.81/32/89 = F91.1/2/9) and 80% ODD (313.81 = F91.3); 54% of patients also had ADHD, which presented as combined type (20%), predominantly inattentive type (8%), or hyperactive/impulsive type (26%) (314.00/01 = F90.0/1/2). Sixteen percent were already receiving ADHD medication prior to the treatment, and no medication change occurred during the treatment. Sixteen percent received psychotherapy from other clinics prior to the study.

### Measures

#### Diagnosis of Conduct Disorders

Symptom criteria for ODD and CD according to ICD-10 and *DSM-IV*, rated by child therapists, were assessed using the semi-structured interview for disruptive behavior disorders (DCL-DBD) of the DISYPS-II (22). The child therapists were requested to rate each of the 25 *DSM-IV*–based items on a four-point Likert scale ranging from 0 (not at all) to 3 (very much). Diagnoses were generated with a diagnostic algorithm. Ratings of 2 or 3 on each item indicate that a *DSM-IV* criterion is fulfilled. The DCL-DBD has been shown to be factorially valid and internally consistent (Cronbach’s α = .68 – .87) (25).

#### Aggressive Behavior–Maintaining Factors

We assessed several maintaining factors of peer- and adult-related aggression of the child at all three time points (pre1, pre2, and post) using the parent and child versions of the Questionnaire for Aggressive Behavior of Children (FAVK-parent and FAVK-child) (26). In accordance with the reading level, only children aged 9 or over were asked to complete the questionnaire, resulting in a smaller sample of patient ratings. The questionnaire consists of four scales that each measure one aggression-maintaining factor: (1) disturbance of social cognitive information processing (FAVK-Soc.-Inf.; 16 items); (2) disturbance of social skills (FAVK-Skills; 12 items); (3) disturbance of impulse control (FAVK-Impulse; 12 items); and (4) disturbance of social interaction (FAVK-Interact; 10 items). Parents/children rated each of the 50 items on a four-point Likert scale ranging from 0 (not at all) to 3 (very much). Mean scores were calculated across all subscales to yield total scores for maintaining factors of peer-related aggression (FAVK-PEER; 25 items) and for maintaining factors of adult-related aggression (FAVK-ADULT; 25 items), with higher scores indicating greater dysfunctionality. The FAVK-PEER total score was defined as the primary outcome measure. Confirmatory factor analyses of parent ratings performed by the test authors confirmed the hypothetical factor model (27). Convergent and divergent validity of this questionnaire has been demonstrated (22, 27). In the present study, the scales showed acceptable to high internal consistencies, with Cronbach’s α ranging from .73 to .96 (parent rating) and from .68 to .89 (child rating) across the three assessment points.

#### Child Aggressive Behavior Problems and Prosocial Behavior

Symptom criteria for ODD and CD according to ICD-10 and *DSM-IV*, as well as prosocial behavior, were assessed *via* parent and child rating of the SCL-DBD of the DISYPS-II (22). Only children aged 11 or older were asked to complete the questionnaire, resulting in a smaller sample of patient ratings. Respondents were requested to rate each of the 37 items on a four-point Likert scale ranging from 0 (not at all) to 3 (very much). We calculated sum scores of the subscale assessing ODD symptoms (SCL-ODD; 9 items) as well as a total score of Disruptive Behavior Disorder (DBD) symptoms (SCL-DBD total; 25 items). Twelve additional items were summed to provide the score of the prosocial behavior subscale (SCL-Prosocial). Higher scores indicate higher levels of problem behavior or prosocial behavior, respectively. The SCL-DBD has been shown to be factorially valid and internally consistent (Cronbach’s α = .69 – .90) (28). In the present study, the scales showed acceptable to high internal consistencies, with Cronbach’s α ranging from .75 to .93 (parent rating) and from .71 to .84 (child rating) across the three assessment points. The SCL-DBD in parent rating was found to be excellent at identifying children with ODD/CD in a community sample (28).

#### Treatment Integrity

Treatment integrity was rated by each therapist after each unit of treatment with ScouT. Moreover, therapists rated the implementation of specific treatment components (e.g., development of a therapeutic relationship with the child, identification of anger cognitions, positive reinforcement of coping cognitions) on a four-point Likert scale ranging from 0 (not implemented) to 3 (very intensively implemented). We calculated means of raw scores (sum of item scores divided by the number of items) as an indicator of the extent of implementation of the treatment components. The internal consistency of the implementation score for the five ScouT units ranged from Cronbach’s α = .95 to .98.

#### Adherence of Patients and Parents

After each session of treatment with ScouT, therapists rated the degree of cooperation of patients and parents during sessions and compliance with therapeutic homework assignments. The adherence scale consisted of five items rated on a four-point scale ranging from 0 (not at all) to 3 (fully true). We calculated the standardized raw score (sum of item scores divided by the number of items) as an indicator of patient adherence and parent adherence. The internal consistency of adherence ratings across all sessions was high both for patient adherence (Cronbach’s α = .98) and for parent adherence (Cronbach’s α = .97).

### Statistical Analyses

#### Analysis of ScouT Treatment Effects

To examine treatment effects of ScouT, we conducted multilevel analyses (29) with the HLM 7 software (30). In the present study, piecewise linear growth models were computed (31, 32), where two different growth rates were taken into account for two different time periods. For the first time period, the changes during the waiting period from pre1 to pre2 were covered by the growth rate β_waiting_. The second time period (treatment period from pre2 to post) was covered by the growth rate β_treatment_. The intercept of the model was treated as random. To enable model identification, the growth rates were fixed. ScouT was considered to have significant treatment effects if change during treatment (growth rate β_treatment_) was significantly larger than change during the waiting period (growth rate β_waiting_). To test β_treatment_ against β_waiting_, contrasts were defined and tested for significance with a χ^2^ test.

To assess the magnitude of the effects for the different outcome measures, Cohen’s *d* effect sizes (33) were calculated for (1) the waiting period ((mean_pre2_ − mean_pre1_)/*SD*_pre1_) and (2) the treatment period ((mean_post_ − mean _pre2_)/*SD*_pre1_). Thus, we divided the differences of the estimated mean values (implied by the model) by the standard deviation at pre1. According to Cohen (33), effect size values ranging from 0.20 to 0.50 are considered as small, from 0.50 to 0.80 as medium, and greater than 0.80 as large.

#### Treatment of Missing Values

As there is no requirement for complete data over occasions in multilevel modeling under the assumption that data are missing at random (34), incomplete cases remained in the analysis. In all other analyses, missing data were imputed by the expectation-maximization (EM) procedure (35) of SPSS (36).

## Results

### Treatment Integrity

The therapists indicated that depending on the specific units, the degree of implementation of specific treatment components was between 2.35 and 2.65 (out of a maximum possible score of 3). The results indicate that across all treatment components and patients, most of the components were predominantly implemented.

### Treatment Adherence

Across all treatment sessions, the standardized patient adherence score was 2.5 (*SD* = 0.41), and the parent adherence score was 2.7 (*SD* = 0.38), indicating high adherence (maximum possible score = 3) for patients and parents.

### Treatment Effects

#### Parent-Reported Outcomes

**Table 1** summarizes the means and standard deviations for all parent-rated outcome measures (all FAVK-parent and SCL-DBD scale scores) at the three assessment points, together with the growth rates for the waiting and treatment period, the χ^2^ values from the contrasts between the two time periods, and the Cohen’s *d* effect sizes.

**Table 1:** Means and standard deviations, results of the multilevel analyses, and Cohen’s d effect sizes for all outcome measures in parent rating at the three assessment points.

Scale	Pre1 *M* (*SD*)	Pre2 *M* (*SD*)	Post *M* (*SD*)	ß_waiting_ (Pre1/Pre2)	ß_treatment_ (Pre2/ Post)	ß_waiting_ vs. ß_treatment_ χ^2^ (*df* = 1)	*d*_waiting_ (Pre1/Pre2)	*d*_treatment_ (Pre2/Post)
FAVK-Parent (*N* = 50)								
SCL-DBD (*N* = 50)								
PEER^a^	1.55(0.47)	1.61(0.42)	0.91(0.60)	0.06	−0.35***	21.17***	0.15	−1.66
ADULT	0.96(0.38)	0.97(0.47)	0.56(0.47)	0.01	−0.21***	6.62**	0.03	−0.88
Soc.-Inf.	1.36(0.52)	1.43(0.44)	0.85(0.58)	0.07	−0.29***	12.90***	0.14	−1.33
Impulse	1.72(0.56)	1.76(0.60)	1.06(0.71)	0.04	−0.34***	9.37**	0.06	−1.14
Skills	1.31(0.56)	1.29(0.62)	0.69(0.63)	−0.02	−0.30***	5.32**	−0.03	−0.97
Interact	0.68(0.41)	0.72(0.45)	0.35(0.33)	0.04	−0.18***	7.32**	0.10	−0.83
ODD	1.83(0.46)	1.83(0.46)	1.12(0.68)	−0.00	−0.35***	10.23***	0.00	−1.55
DBD-Total	0.91(0.42)	0.91(0.42)	0.51(0.32)	0.00	−0.20***	6.66**	0.00	−0.94
Prosocial	1.50(0.39)	1.60(0.45)	1.90(0.49)	0.10	0.15***	0.35	0.26	0.66

FAVK-parent, Questionnaire for Aggressive Behavior (parent rating); PEER, total score for factors of peer-related aggression; ADULT, total score for factors of adult-related aggression; Soc.-Inf., disturbance of social cognitive information processing; Impulse, disturbance of impulse control; Skills, disturbance of social skills; Interact, disturbance of social interaction; SCL-DBD, Symptom Checklist for Disruptive Behavior Disorder; ODD, oppositional defiant disorder; DBD-Total, Disruptive Behavior Disorder Total score; M, mean; SD, standard deviation; df, degrees of freedom; d, effect sizes for both periods: differences of the estimated mean values (implied by the model) divided by the standard deviation to pre; d_waiting_ = (mean_pre2_ − mean_pre1_)/SD_pre1_ and d_treatment_ = (mean_post_ − mean_pre2_)/SD_pre1_); β, growth rate (change during period).

a Primary outcome measure.

** p < 0.01, *** p < 0.001.

For the waiting period (pre1 to pre2), the growth rates (ß_waiting_) were not significantly different from zero for the primary outcome measure (FAVK-PEER) or for any of the secondary outcome measures, indicating no significant change during the waiting period. For the treatment period (pre2 to post), the growth rates (ß_treatment_) of the primary outcome measure (FAVK-PEER) and all secondary outcome measures differed significantly from zero. These results indicate a decrease in child problem behavior and an increase in competencies during treatment from pre2 to post. The contrasts of both growth rates (ß_waiting_ vs. ß_treatment_) were significant for the primary outcome measure (FAVK-PEER) and for all secondary outcome measures except for the SCL-Prosocial subscale. The significant contrasts indicate a greater decrease in behavior problems and problem-maintaining factors during the treatment period than during the waiting phase.

With the exception of SCL-Prosocial (*d* = .26), the effect sizes were all below or equal to *d* = 0.15 for the waiting period. For the treatment period, effect sizes for the primary outcome measure (FAVK-PEER), the other FAVK subscales, and oppositional behavior problems (SCL-ODD and SCL-Total) were large (*d* = −0.83 to *d* = −1.66). The effect size for increase in competencies was medium.

**Figure 2:**
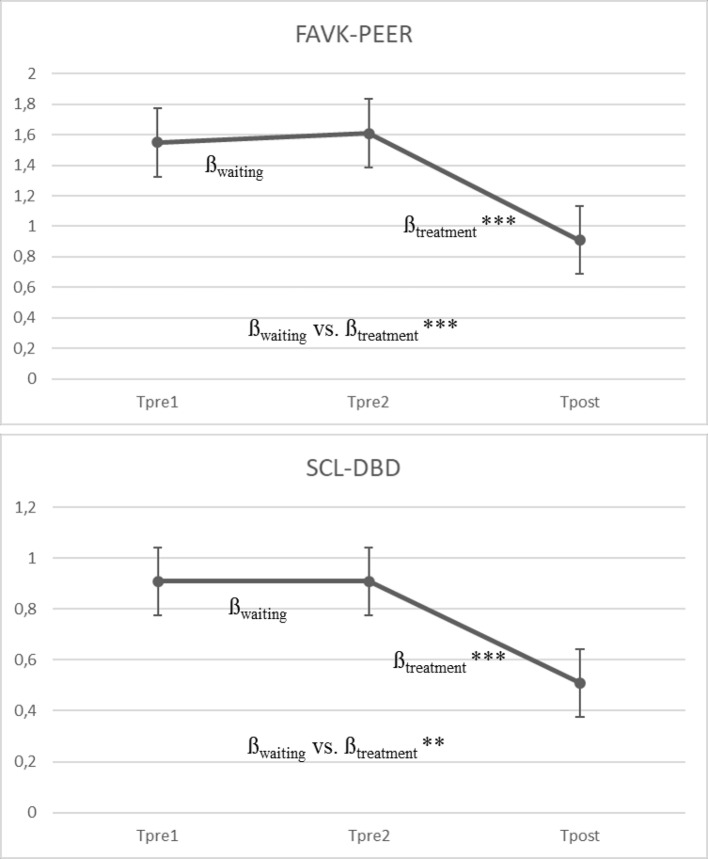
Mean growth curve for FAVK-PEER and SCL-DBD based on the multilevel model (*N* = 50). β_waiting_ = slope of the waiting period (pre1/pre2); β_treatment_ = slope of the treatment period (pre2/post). ^***^ p < .001, ^**^ p < .01. Measures: FAVK-PEER, total score for maintaining factors of peer-related aggression. SCL-DBD, Symptom Checklist for Disruptive Behavior Disorder.

**Figure 2** presents the results of the multilevel models for the primary outcome measure of peer-related aggressive behavior (FAVK-PEER) and for the SCL-DBD total score.

#### Child-Reported Outcomes

**Table 2** presents the means and standard deviations for all self-rated outcome measures (all FAVK-child scale scores and SCL-DBD scale scores) for the subsample of children aged 9 years or older (FAVK; *n* = 35)/11 years or older (SCL-DBD; *n* = 15) at the three assessment points, together with the growth rates for the waiting and treatment period and the χ^2^ values from the contrasts between the two time periods as well as the Cohen’s *d* effect sizes. For the waiting period (pre1 to pre2), the growth rates (ß_waiting_) were not significantly different from zero for all outcome measures, indicating no significant change during the waiting period. For the treatment period (pre2 to post), the growth rates (ß_treatment_) differed significantly from zero for the primary outcome measure (FAVK-PEER) and for all secondary outcome measures except for the SCL-Prosocial subscale. These results indicate a decrease in child problem behavior during treatment from pre2 to post. However, the contrasts of both growth rates (ß_waiting_ vs. ß_treatment_) were only significant for the primary outcome measure (FAVK-PEER), indicating a greater decrease during the treatment period than during the waiting phase. For child behavior problems, the effect sizes were all below or equal to *d* = 0.30 for the waiting period and moderate to large for the treatment period (*d* = .47 to *d* = −1.34).

**Table 2:** Means and standard deviations, results of the multilevel analyses, and Cohen’s d effect sizes for all outcome measures in self-rating at the three assessment points.

Scale	Pre1 *M* (*SD*)	Pre2 *M* (*SD*)	Post *M* (*SD*)	ß_waiting_ (Pre1/Pre2)	ß_treatment_ (Pre2/Post)	ß_waiting_ vs. ß_treatment_ χ^2^ (*df* = 1)	*d*_waiting_ (Pre1/Pre2)	*d*_treatment_ (Pre2/Post)
FAVK-Child (*n* = 35; 9–12 years old)								
SCL-DBD (*n* = 15; 11–12 years old)								
PEER	1.05(0.43)	1.01(0.44)	0.55(0.39)	−0.04	−0.23***	3.77*	−0.09	−1.05
ADULT	0.52(0.34)	0.46(0.40)	0.27(0.32)	−0.06	−0.09**	0.16	−0.18	−0.47
Soc.-Inf.	0.86(0.38)	0.82(0.48)	0.49(0.36)	−0.05	−0.16***	1.53	−0.13	−0.68
Impulse	1.11(0.55)	0.95(0.60)	0.48(0.50)	−0.11	−0.05***	0.25	−0.30	−0.78
Skills	0.69(0.48)	0.66(0.49)	0.32(0.46)	−0.03	−0.17***	1.63	−0.06	−0.71
Interact	0.50(0.38)	0.53(0.40)	0.33(0.42)	0.03	−0.10**	1.68	0.08	−0.51
ODD	1.33(0.42)	1.20(0.41)	0.65(0.43)	−0.13	−0.27***	0.71	−0.30	−1.34
DBD-Total	0.60(0.24)	0.54(0.19)	0.30(0.15)	−0.06	−0.12***	0.44	−0.27	−1.25
Prosocial	1.95(0.41)	1.91(0.47)	2.12(0.38)	−0.04	0.11	0.93	−0.11	0.47

^*^ p < 0.05, ^**^ p < 0.01, ^***^ p < 0.001.

### Clinical Significance

In line with the study inclusion criteria, all patients had a high symptom score (Stanine ≥ 7) before treatment at the pre1 and pre2 assessments on the parent-rated SCL-DBD total score. At the post-assessment, 46% of patients (n = 23) had dropped below this cutoff (indicating normalization), while 54% (n = 27) of patients remained in the clinical range.

## Discussion

To the best of our knowledge, the present study is the first to evaluate the effects of an individualized computer-facilitated social skills training program for clinically referred children with peer-related aggressive behavior and a diagnosis of ODD/CD. We assessed the effects of the primarily child-focused, individually tailored treatment (consisting of 16 child treatment sessions and 2 parent psychoeducation sessions) using a within-subject control group design, which compared the course of symptoms during an 8-week waiting period with the course during the subsequent 16-week treatment period.

We found large treatment effects on the primary outcome measure of maintaining factors of peer-related aggression (FAVK-PEER) as rated by the parents as well as on secondary outcomes in parent rating (maintaining factors of adult-related aggression, disturbance of social cognitive information processing, social skills, impulse control, and social interactions) and on parent-rated symptoms of ODD as defined by ICD-10 and *DSM-IV*. There was also a large treatment effect on the maintaining factors of peer-related aggression as rated by the child, which also differed significantly from changes in the waiting phase. For the other self-reported outcomes, we observed moderate to large effect sizes but did not find a significant effect during the treatment phase as compared to the waiting phase.

The current study extends the results of the—to our knowledge—only other study on the effects of a computer-facilitated interactive social skills training program, which was conducted in boys with externalizing behavior problems (8). However, the sample of the analysis by Fenstermacher et al. (8) was restricted to four boys and used only analogue role-play assessments to measure treatment outcome.

Another study by our research group on the effects of individualized treatment of peer-related aggression in boys using a similar treatment rationale as in ScouT, but without computer facilitation, and a similar within-subject design to analyze treatment effects (13) reported somewhat higher effect sizes during treatment. However, the patients in the present study attended fewer therapy sessions than those in the aforementioned study, and parental involvement was also lower in the present study. In view of this, it is impressive that changes during treatment with ScouT are mainly in the large range. This finding might be related to the use of a computer-facilitated treatment program in contrast to a traditional therapy setting. However, this study does not provide a direct comparison between a treatment including a digital component and an intervention without such a component. The clinical impression was that the use of these technologies was attractive for children at the age of 6 to 12 years and that this may have contributed to a higher treatment motivation and patient adherence, which may in turn have led to a better outcome. Indeed, the patient adherence scores were slightly higher than in our previous study (13). However, adherence scores in both trials were high in general, meaning that ceiling effects cannot be ruled out when comparing the two trials. The hypothesis of an increased patient motivation and adherence also matches our clinical impression. It is also plausible that the computer-facilitated therapy manual of ScouT led to an enhanced treatment integrity, which might likewise have resulted in stronger treatment effects on the various outcomes. Indeed, treatment integrity scores in the present study were higher than those in our previous trial in a traditional therapy setting (2.35–2.65 vs. 1.5–2.2 out of a maximum score of 3, respectively). At this point, however, it should be noted as a limitation that treatment integrity was only rated by therapists, and an additional independent rater would have been useful.

Our findings regarding the parent ratings are consistent with results of meta-analyses (5, 7) that demonstrated the efficacy of child-focused or child- and parent-focused interventions for patients with aggressive behavior problems. It is noteworthy that we found large effect sizes for the treatment with ScouT, while McCart et al. (7) reported—on average —only small effects for child-centered treatment of antisocial behavior problems. This discrepancy may be due to the therapy setting in our trial: Unlike most other trials, the intervention was conducted in individual treatment sessions, which may be more intensive and tailored to the specific needs and problems as well as the individual problem-maintaining factors of the child. ADHD medication cannot contribute to the treatment effects, since no medication change occurred during the treatment. In contrast, most other studies tend to use standard problem situations in child group sessions, and the treatment is often not adapted to the specific factors that maintain the aggressive behavior. In a recent meta-analysis of long-term effects of outpatient treatment in children and adolescents with conduct problems, Fossum et al. (37) showed that individual treatments resulted in larger changes in aggressive behavior as compared to group treatments.

Notably, the studies reported in the meta-analysis by McCart et al. (7) compared child-centered treatment of antisocial behavior with no treatment or wait-list control groups. In contrast, we report on a within-subject control design, which certainly constitutes a limitation to our findings, as the design is less rigorous than a randomized controlled trial. Within-subject designs have specific advantages and disadvantages (38): Advantages include the reduction of error variance and increase of statistical power, since each participant is used as his/her own control. The present analysis is part of a larger randomized controlled trial, in which the treatment with ScouT will be compared to an active control group (Goertz-Dorten et al., in prep.). Because of the comparison to an active control group in the randomized controlled trial (RCT), the research question that may be answered by a between-subjects analysis differs from that of the analysis that is reported here, especially with respect to the effect sizes: in our within-subjects approach, we can say something about the effects of ScouT in comparison to no treatment, whereas the comparison with another active treatment gives information about the superiority of ScouT to a different therapeutic approach.

The current study is one of the few studies to also assess treatment outcome as rated by children. In the subsamples of children aged 9 years/11 years or older, self-reported problem-maintaining factors of peer-related and adult-related aggression and of ODD symptoms as defined by ICD-10/*DSM-IV* were also significantly reduced during treatment, as reflected in the significant growth rates from pre2 to post-treatment. However, when comparing the symptom courses during the waiting period and the treatment period, a treatment effect could only be found for maintaining factors of peer-related aggression. Similar results emerged in our previous study (13). The non-significant effects on most child-based measures in both studies may be partly caused by the reduced statistical power due to the smaller sample sizes.

Child self-report information may be especially important in the assessment and treatment of peer-related aggression because parents or teachers may observe only a small part of conflict situations with peers. However, the reliability and validity of the ratings may be questionable. At pre1, we observed much lower scores in self-rating than in corresponding scales in parent rating. This might indicate a tendency for children to underreport the intensity of the problem behavior. Additionally, the lower scores at the beginning of treatment may reduce the potential for symptom reduction during treatment. Another reason for the differences in significant effects in parent and child rating may be that the child report is less sensitive to treatment change.

Despite the aforementioned limitations in interpreting the results, the assessment of patient-reported outcome measures constitutes an improvement compared to most other studies in this age range. Nevertheless, a further third-party rating (especially peers, but also clinician, teacher) would have been useful. The results primarily reflect the perception of the parents, who also participated in the treatment, meaning that an effort justification effect cannot be precluded. However, as the treatment was primarily child-centered (with only 2 parent sessions in addition to the 16 child sessions), and as other studies have shown that the effects of parent training cannot necessarily be attributed to effort justification of the parent (39), we find it unlikely that our treatment effects are solely the result of a parental response bias.

Further limitations of the present study should be mentioned. First, only 8% of the sample was female. Therefore, as in most published studies, the results may be primarily valid for boys. Second, the sample was predominantly Caucasian and recruited from an urban area in Germany, which further limits the generalizability of the findings. Third, as the present analysis did not compare the computer-assisted treatment with alternative interventions or treatment as usual, it was not possible to evaluate the benefit compared to other treatments. Further analyses are currently being conducted to answer this question. Fourth, this quasi-experimental within-subject comparison did not control for all confounding variables. Therefore, other factors may have influenced the course during treatment. However, by comparing growth rates during the waiting period with growth rates during treatment, it was possible to establish a within-subject control, and the differences in the length of the two periods were corrected. Moreover, the interventions were not conducted in parallel for all patients, but took place during the course of 2 years, and no substantial differences were found in the percentage of non–school days during the waiting phase compared to the treatment phase. Therefore, seasonal effects (e.g., school holidays) could be ruled out as confounding factors. Fifth, as follow-up data are lacking in the present analysis, the stability of the treatment effects is unknown, although the long-term effects of this intervention are currently being assessed. Finally, the principal investigators of the study (AG-D, MD) are also authors of the treatment program ScouT. A replication of the study by an independent research group would be appreciated in order to rule out researcher allegiance.

Besides an independent replication of our findings and a direct comparison of the effects of ScouT with those obtained in a traditional individual therapy setting, our study gives further implications for future research. As our trial was conducted in an outpatient unit for children with ODD/CD under rigorous research conditions, the treatment probably differed from treatment as usual. Accordingly, it is necessary to conduct a trial under routine care conditions, because results of effectiveness trials are often less positive than those of efficacy trials (40, 41).

Despite the aforementioned limitations and the necessity for further research, our study demonstrates that a computer-facilitated individualized treatment of peer-related aggression may be an effective treatment, leading to similar outcomes to those found in traditional treatment, and with less therapeutic effort or time required. Moreover, the use of technology in child and youth psychotherapy is a promising way to enhance treatment motivation and patient adherence, which is likely to result in better outcomes.

## Data Availability

The datasets generated for this study are available on request to the corresponding author.

## Ethics Statement

This study was carried out in accordance with the recommendations of the ethics committee of the University Hospital, Cologne with written informed consent from all subjects. All subjects gave written informed consent in accordance with the Declaration of Helsinki. The protocol was approved by the ethics committee of the University Hospital, Cologne.

## Author Contributions

AG-D and MD developed the Intervention, designed the treatment study, analyzed the data, and did the writing. MG, KD, AH, LS, and BP conducted the treatments, collected the data, managed and analyzed the data and contributed to the text.

## Funding

The study received financial support from the School of Child and Adolescent Cognitive Behavior Therapy at the University Hospital Cologne.

## Conflict of Interest Statement

AG-D and MD are authors of the evaluated treatment manual and of the rating scales used for evaluation, for which they receive royalties from publishing companies.

The remaining authors declare that the research was conducted in the absence of any commercial or financial relationships that could be construed as a potential conflict of interest.
